# Osseointegration of a 3D Printed Stemmed Titanium Dental Implant: A Pilot Study

**DOI:** 10.1155/2017/5920714

**Published:** 2017-11-19

**Authors:** James Tedesco, Bryan E. J. Lee, Alex Y. W. Lin, Dakota M. Binkley, Kathleen H. Delaney, Jacek M. Kwiecien, Kathryn Grandfield

**Affiliations:** ^1^Department of Materials Science and Engineering, McMaster University, Hamilton, ON, Canada; ^2^School of Biomedical Engineering, McMaster University, Hamilton, ON, Canada; ^3^Department of Materials Science and Engineering, Northwestern University, Evanston, IL, USA; ^4^School of Integrated Science, McMaster University, Hamilton, ON, Canada; ^5^Department of Pathology and Molecular Medicine, McMaster University, Hamilton, ON, Canada; ^6^Department of Clinical Pathomorphology, Medical University of Lublin, Lublin, Poland

## Abstract

In this pilot study, a 3D printed Grade V titanium dental implant with a novel dual-stemmed design was investigated for its biocompatibility in vivo. Both dual-stemmed (*n* = 12) and conventional stainless steel conical (*n* = 4) implants were inserted into the tibial metaphysis of New Zealand white rabbits for 3 and 12 weeks and then retrieved with the surrounding bone, fixed, dehydrated, and embedded into epoxy resin. The implants were analyzed using correlative histology, microcomputed tomography, scanning electron microscopy (SEM), and transmission electron microscopy (TEM). The histological presence of multinucleated osteoclasts and cuboidal osteoblasts revealed active bone remodeling in the stemmed implant starting at 3 weeks and by 12 weeks in the conventional implant. Bone-implant contact values indicated that the stemmed implants supported bone growth along the implant from the coronal crest at both 3- and 12-week time periods and showed bone growth into microporosities of the 3D printed surface after 12 weeks. In some cases, new bone formation was noted in between the stems of the device. Conventional implants showed mechanical interlocking but did have indications of stress cracking and bone debris. This study demonstrates the comparable biocompatibility of these 3D printed stemmed implants in rabbits up to 12 weeks.

## 1. Introduction

Bone-anchored implants have been a standard treatment for edentulism since the mid-1980s, after Per-Ingvar Brånemark demonstrated the successful osseointegration of a titanium dental implant placed in human patients [1]. However, failure rates for clinical use dental implants range between 3 and 8% depending on the implant design and/or patients' health factors [2–5]. Although this appears to be a rather successful procedure, an epidemiological study reported 1200 emergency department visits due to dental implant failures from 2008 to 2010 in the US alone [6], signifying the continued burden of edentulism on the healthcare system. As such, methods to improve the clinical outcomes of dental implants are still actively pursued.

Due to its bone-bonding or osseointegrative ability, mechanical and chemical properties, and overall biocompatibility, titanium and titanium alloys have long been the dental implant material of choice [7–9]. Recently, considerable emphasis has been placed on surface treatment of implants, where surface roughness and texture modifications have been shown to facilitate bone integration and cellular activity via microscale and nanoscale features [10–13]. In addition, a range of surface coatings, such as calcium phosphate, magnesium, and titania, have been explored with the intent of encouraging faster osseointegration [14–16]. While it is known that implant geometry can change the response of the bone-implant interface under loading [17], conventional machining processes have traditionally limited implant morphologies to conical- and screw-like designs. However, with the technological advances in additive manufacturing, 3D printing of titanium and titanium alloys for new and innovative implant geometries are now possible. Additive manufacturing techniques, such as direct metal laser sintering (DMLS) and electron beam melting (EBM), are processes that can create three-dimensional metallic constructs by selectively melting metal powder in a layer-by-layer fashion. These techniques are capable of creating complex porous features [18–20] and an inherent surface roughness, as the melted powder droplets solidify on the object surface. Current use of this technology for implant manufacturing has focused on creating open pore networks to mimic the trabecular bone, showing improved cellular activity and greater bone ingrowth in both rabbit and sheep models [21–23].

In this pilot study, the osseointegration of a dental implant with a novel dual-stemmed shape, enabled by DMLS, was assessed for the first time. We present complementary histology, high-resolution X-ray, and electron microscopy analyses to investigate the bone-implant interface.

## 2. Materials and Methods

### 2.1. Implants

**Figure 1:**
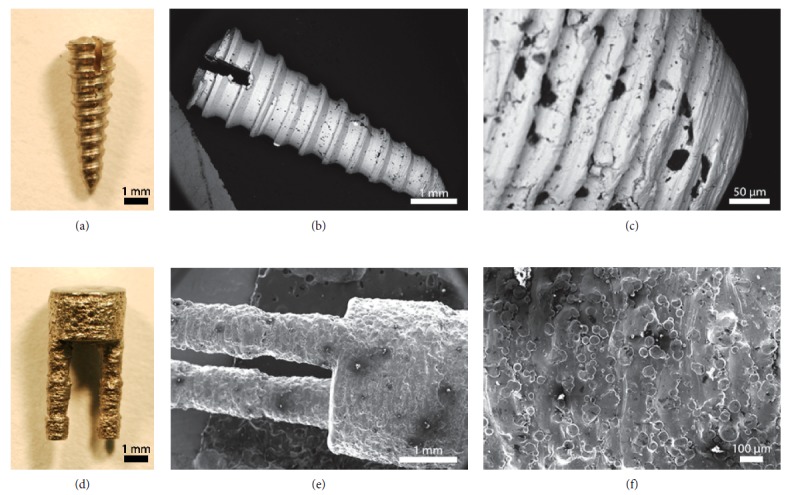
(a) Photo of control conical implant (Ø = 2 mm, *l* = 6 mm). (b, c) SEM images of control surface. (d) Photo of dual-stemmed (SIT) implant (Ø = 3 mm, *l* = 6 mm). (e, f) SEM images of SIT implant surface. At higher magnification, it is clear that the 3D printed surface (f) retains characteristic surface features representative of the powders used in its production and has a much higher roughness than conventionally machined implants (c).

Twelve dual-stemmed implants (herein referred to as the SIT implant) were produced via DMLS using an EOSINT M 280 (EOS GmbH, Krailling, Germany) with Ti6Al4V powder and were received from Stemmed Implant Technologies Inc. (Niagara Falls, Canada). Implants were 3 mm in diameter, with 1 mm diameter stems. In preparation for implantation, implants were cut into 6 mm in length, briefly sandblasted with 70 psi and a 90% glass bead/10% Al_2_O_3_ media, and autoclaved. Final implants consisted of a body and stems, both 3 mm in length (Figure 1). Four conical stainless steel mini-implant screws, with a tapered body and maximum of 2 mm diameter, were used as controls. The control implants were received from Stemmed Implant Technologies Inc., cut into a 6 mm length to match the length of the stemmed implants (Figure 1), and autoclaved for sterilization prior to the implantation procedure. At higher magnification, it is clear that the 3D printed surface (f) retains characteristic surface features representative of the powders used in its production and has a much higher roughness than conventionally machined implants (Figures 1, 1, 1 and 1).

### 2.2. Implant Placement and Retrieval

**Figure 2:**
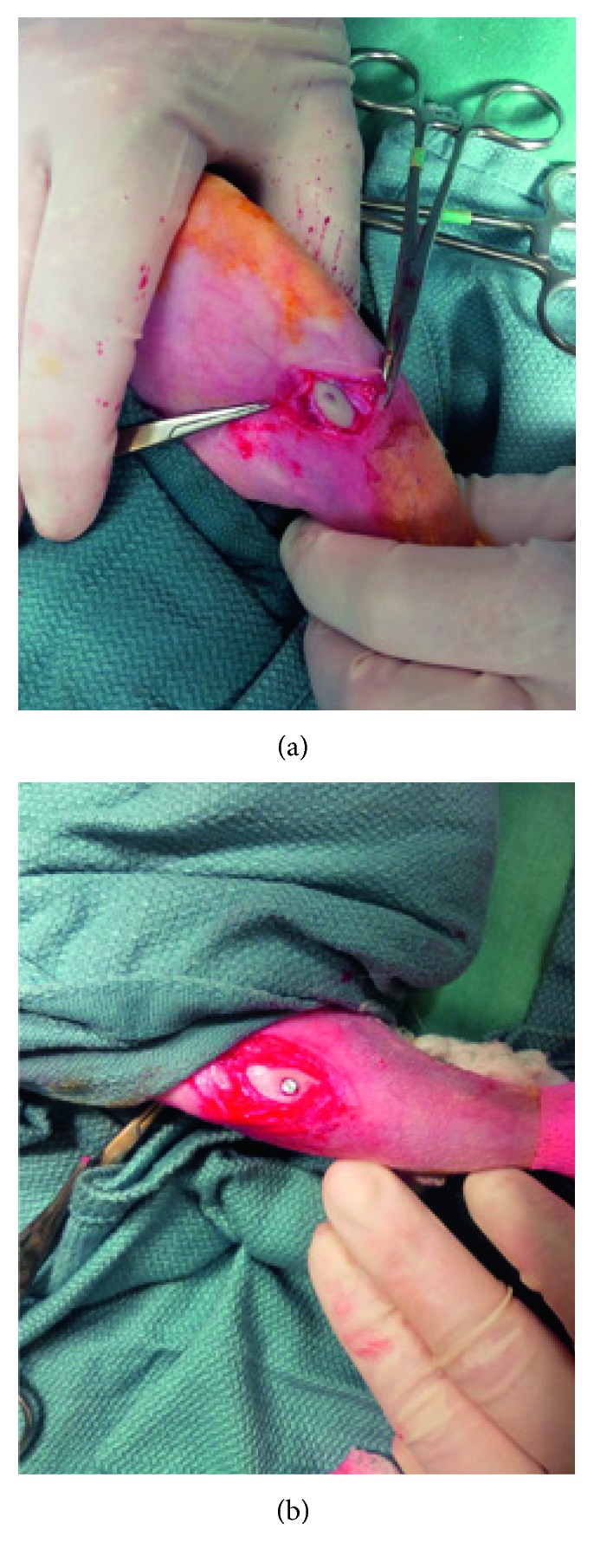
Cranial medial surgical approach parallel to the tibial crest. (a) Slow-rotating drill used to create pilot holes perpendicular to the bone crest. (b) SIT implants were pressed to fit into place until flush with bone crest. Control implants were screwed into place (not shown).

Eight skeletally mature female-specific pathogen-free New Zealand white (NZW) rabbits (Charles River, Toronto, Canada) weighing between 3 and 4 kg were housed at the Central Animal Facility (CAF) at McMaster University. Animal experiments were carried out under ethical approval (AUP 14-12-54) from the McMaster Animal Research Ethics Board. The day prior to surgery and over the following four days, all rabbits received 10 mg/kg enrofloxacin (Baytril®, Bayer, Leverkusen, Germany) to prevent infection. During surgery, the rabbits were induced with xylazine, ketamine, and acepromazine, intubated, and placed on isoflurane gas 2-3% inhalation with oxygen. Buprenorphine was administered at 0.5 mg/kg subcutaneously to prevent pain and readministered every 12 hours for 48 hours postoperatively. The surgical method was a cranial medial approach parallel to the tibial crest with a slow-rotating drill and irrigation with saline (Figure 2). The implant bed therefore consisted of cylindrical holes through the cortical bone slightly smaller than the diameter of the implants. One implant of each type was inserted into the tibial metaphysis of each rabbit, where stemmed implants were pressed to fit and conical implants were screwed in until flush with the bone crest. Incisions were closed with a layer of 4-0 Vicryl® (Ethicon, Inc., Somerville, USA), and the skin was closed with stainless steel wound clips. Animals were then randomly split into two groups: one provided a 3-week healing period, and the other, 12 weeks. After the healing period, rabbits were euthanized by overdose of barbiturate. However, one animal at each time point was perfused with 2% glutaraldehyde in 0.1 M sodium cacodylate buffer solution, and bone-implant sections were removed for decalcification with EDTA for histology.

Implants with surrounding bone tissue were collected and prepared into implant-bone blocks following the methodology for preparing undecalcified bone outlined by Donath and Breuner [24]. Approximately 2 cm × 2 cm bone blocks containing the implants were fixed in a solution of 1% glutaraldehyde and 1% paraformaldehyde in 0.1 M sodium cacodylate buffer for 7–10 days and subsequently dehydrated in a graded series of ethanol, followed by embedding in LR white acrylic resin for the 3-week specimens and Embed-812 epoxy resin for the 12-week specimens. Blocks were longitudinally sectioned using an Isomet® low-speed saw (Buehler, Lake Bluff, USA) and a diamond wafer blade to reveal the bone-implant interface.

### 2.3. Histology

Rabbits were overdosed with 65 mg/kg body weight sodium pentobarbital I.V. and perfused via the left cardiac ventricle with 1 L lactated Ringer's solution followed by 1 L formalin (10% paraformaldehyde in phosphate buffer, pH 7.2). The fragment of the tibial bone with the metal implant was carefully removed, postfixed in formalin for two days, and then placed in formalin supplemented with 4% EDTA (pH 7.2) for demineralization. Demineralizing solution was exchanged once per week over nine months. The metal implants were carefully removed from the soft bone, and the bone was sectioned for analysis at the site of the implants. The sections were dehydrated with a series of graded concentrations of ethyl alcohol (50–100%) and xylene, embedded in paraffin wax, cut into 5 µm thick, mounted onto glass slides, and stained with both hematoxylin and eosin. The histological analysis was performed under an Eclipse 50i light microscope (Nikon, Tokyo, Japan).

### 2.4. Microcomputed Tomography

Visualization of whole implant-bone ingrowth was achieved using a SkyScan 1172 (Bruker, Billerica, USA) with a 100 kV X-ray beam, aluminium-copper filter, 2.3 µm–2.6 µm pixel size, and 0.3–1° rotation step. NRecon and CTAn software (Bruker, Billerica, USA) were used to reconstruct and visualize the 3D volumes. The length of bone growth was measured using the image processing and analysis software ImageJ (National Institutes of Health, Bethesda, USA).

### 2.5. Scanning and Transmission Electron Microscopy

Longitudinal implant-bone blocks were sputter coated with gold and imaged with a JSM-6610LV (JEOL Ltd., Tokyo, Japan) scanning electron microscope (SEM) at an accelerating voltage of 10 kV. Backscattered electron (BSE) images with compositional contrast enabled identification of regions of new bone growth along the implant length.

**Figure 3:**
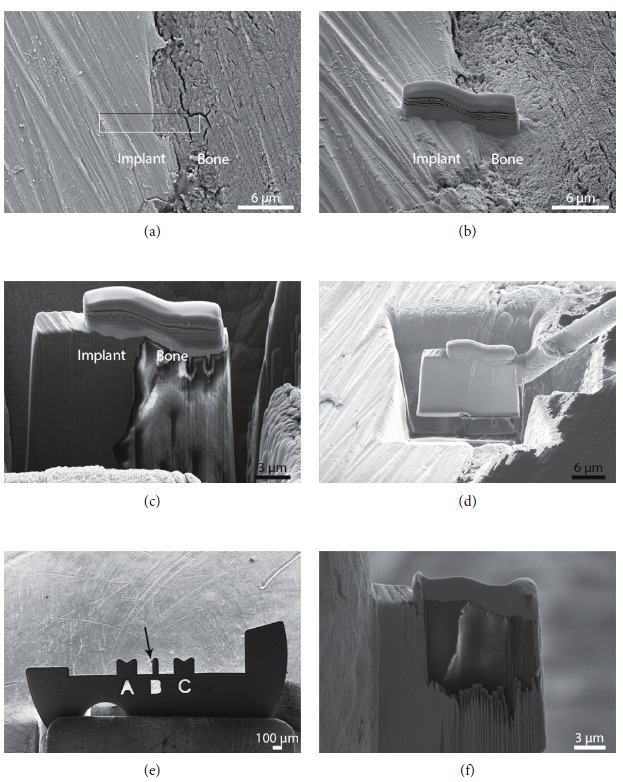
TEM sample preparation of a SIT 12-week bone-implant interface using FIB. (a) Selection of ROI. (b) Deposition of protective carbon layer over ROI. (c) Rough milling of the material surrounding the ROI. (d) Lift-out of the lamella containing the ROI via an in situ micromanipulator. (e) Attachment of the lamella on the copper TEM grid (arrow indicates sample location). (f) Cross-sectional view of the electron transparent sample following final thinning.

Transmission electron microscopy (TEM) specimens were prepared using an in situ lift-out method (Figure 3) on a NVision 40 (Carl Zeiss GmbH, Germany), a dual-beam instrument comprising a focused ion beam (FIB) milling instrument and a Schottky field emission gun (FEG) filament SEM. Due to implant-bone separation caused during retrieval and sample preparation, an intact bone-implant specimen was not possible. However, the interface between old and new bone was successfully prepared for high-resolution analysis. TEM images were captured using a Titan 80-300 (FEI, Oregon, USA) operated at 300 kV with a high-angle annular dark-field detector.

## 3. Results

### 3.1. Histology

**Figure 4:**
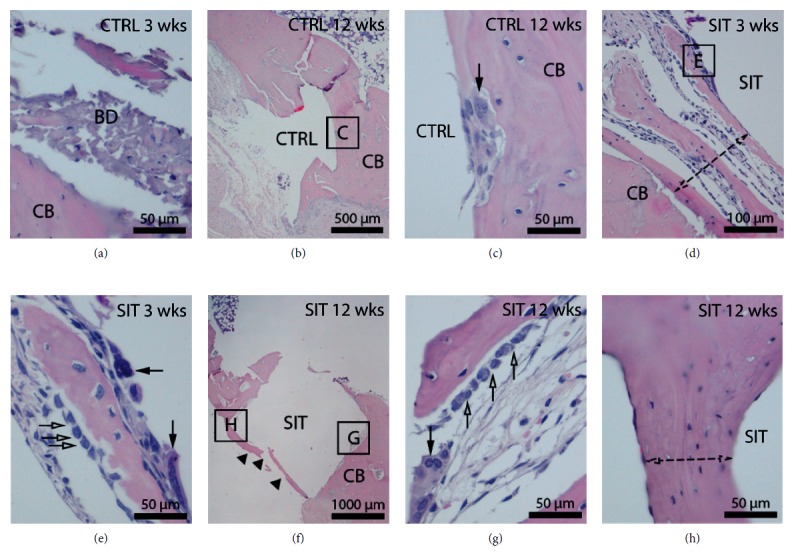
H&E staining highlights histological changes in the tibial bone after implantation of the SIT implant (d, e, f, g, h) or control implant (a, b, c) for a duration of 3 weeks (a, d, e) or 12 weeks (b, c, f, g, h). The metal implants have been removed. (a) At 3 weeks post implantation, bone debris is present between the control implant and cortical bone. (b) At 12 weeks post implantation, the cortical bone surrounding the control implant has active bone remodeling at the bone-metal interface (c) which involves osteoclasts (arrow). However, the cortical bone is being actively remodeled (double-headed arrow) around the site of the SIT implant (d) after 3 weeks. At higher magnification (e), multinucleated osteoclasts (black arrows) and hypertrophied osteoblasts (white arrows) participate in bone remodeling. (f) At 12 weeks post implantation, the SIT implant is encased in the cortical bone (arrowheads) which at higher magnification (h) has the morphology of a mature bone (double-headed arrow). The remodeling of the bone surrounding the SIT implant is still active (g) and involves osteoclasts (black arrow) and hypertrophied osteoblasts (white arrows). CTRL = control, SIT = stemmed implant, CB = cortical bone, and BD = bone debris.

Histological analysis of both SIT implants and control screw implants was completed after 3 and 12 weeks of implantation to determine the cellular activity and remodeling behaviour of the bone tissue with the implanted devices (Figure 4). For both the SIT and control groups, implants resided primarily in the cortical bone. Following extraction of the control implant after 3 weeks, bone debris was present between the implant and the cortical bone (Figure 4). This is in contrast to the cortical bone surrounding the SIT implant after 3 weeks, where there was no debris and the presence of multinucleated osteoclasts and hypertrophied osteoblasts suggested that the bone was being actively remodeled around the implant (Figures 4 and 4). After 12 weeks of implantation, both the control-implanted (Figure 4) and SIT-implanted (Figure 4) rabbits were observed to have active bone remodeling at the bone-implant interface. The control implants showed active bone remodeling involving osteoclasts (Figure 4), meanwhile, the SIT implant was completely encased in the cortical bone, with a layer of bone forming over its surface. The remodeling of the bone was still active but showed the morphology of more mature bone (Figure 4).

### 3.2. Microcomputed Tomography

**Figure 5:**
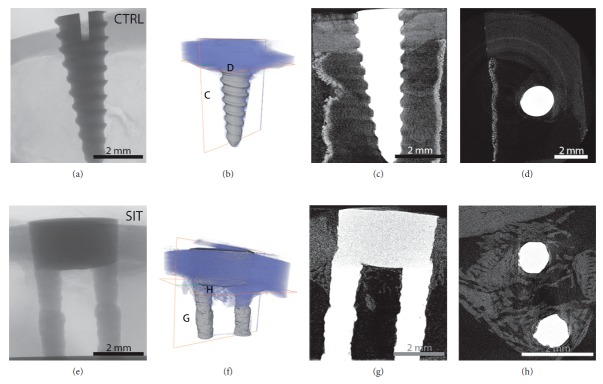
Micro-CT analysis following 3-week implantation of CTRL (left column) and SIT (right column) implants. (a, e) Radiographs of each implant type. (b, f) 3D visualization of the implant and bone (purple) with orthogonal planes labelled, (c, d, g, h) corresponding orthoslices from (b, f) where new bone formation (appears lighter in contrast) is noted. Both implants showed bone conduction down the implants from the cortical bone crest, while the SIT implant also showed new bone formation between the implant stems.

**Figure 6:**
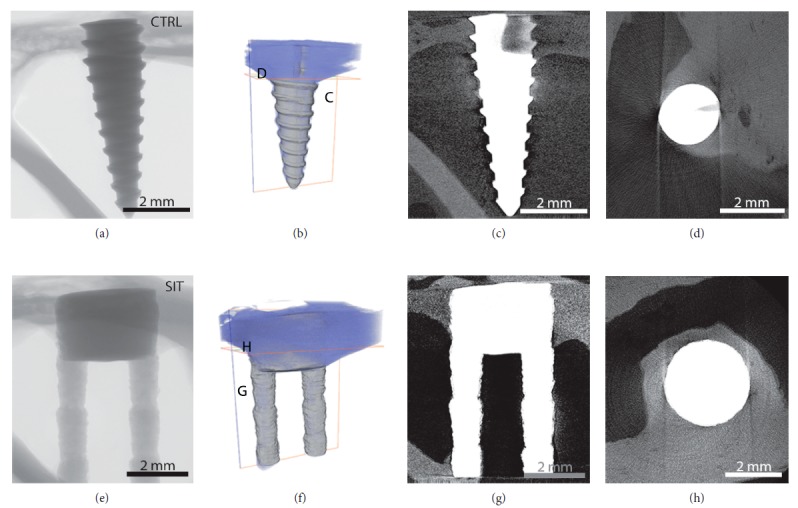
Micro-CT analysis following 12-week implantation of CTRL (left column) and SIT (right column) implants. (a, e) Radiographs of each implant. (b, f) 3D visualization of the implant and bone (purple) with orthogonal planes labelled, (c, d, g, h) corresponding orthoslices from (b, f) where the new bone has matured and is of equal intensity to the preexisting bone. Both implants showed bone conduction down the implants from the cortical bone crest; however, bone growth between the stems was not noted in this particular specimen.

**Figure 7:**
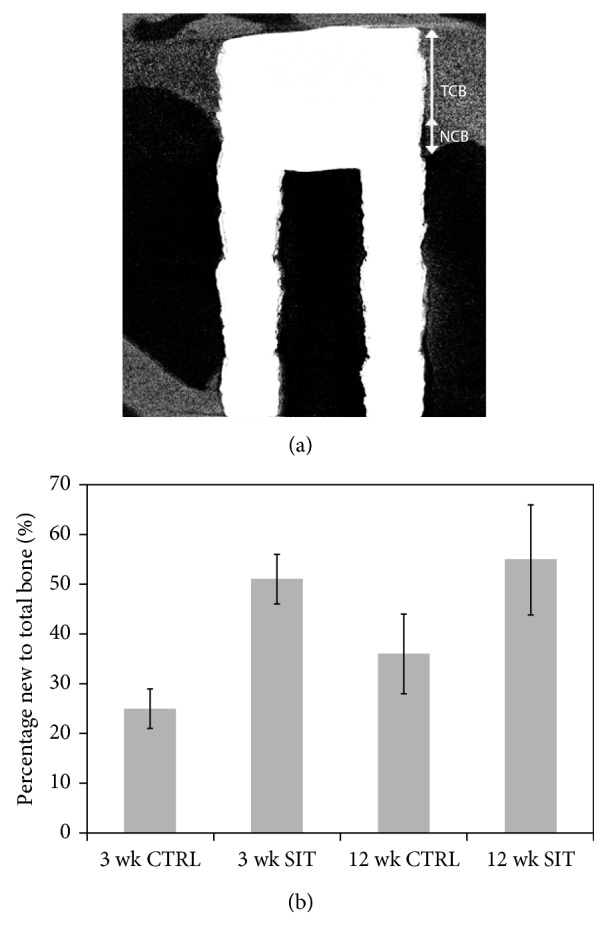
(a) Representative micro-CT orthoslice of the SIT device, indicating new cortical bone growth (NCB) and total cortical bone (TCB) from the coronal surface after implantation. (b) Graphical comparison of new bone formation under the cortical bone crest to total bone crest height observed after 3- and 12-week implantation for control and SIT implants.

Prior to sectioning for SEM, the entire implant-bone blocks of the SIT and control implants were imaged by microcomputed tomography (µ-CT). Radiographs of both implant types revealed the top portion of the implants to be surrounded by the cortical bone with the remainder of the implant located in the medullary cavity (Figures 5, 5, 6, and 6). The SIT and control implants were shown to have new bone growing from the preexisting cortical bone, down and around each implant surface and into the medullary cavity, after 3 and 12 weeks (Figures 5 and 6). Three-dimensional renderings (Figures 5, 5, 6, and 6) of both implants provided a holistic perspective of the entire implant and surrounding bone volume. The new bone growth around the implants is simultaneously visualized with the growth down the implant length. The location of the reconstructions shown in Figures 5 and 6 is represented in the 3D renderings by the corresponding cross-sectional and longitudinal planes. At 3 weeks, the new bone is in an immature state, identified by lighter contrast and porous structure when compared to the preexisting cortical bone for both the control (Figures 5 and 5) and SIT (Figures 5 and 5) implants. Qualitatively, through 12 weeks, the new bone appeared to have developed into mature or remodeled cortical bone with higher levels of mineralization, as a result of the similar contrast and density of the new and old cortical bone for both implant types (Figures 6, 6, 6 and 6). The longitudinal sections of the control (Figures 5 and 6) and SIT (Figures 5 and 6) implants showed a difference in the extent of bone growth extending from the bone crest down the length of the implant at both time points. The new bone accounted for 25% and 50% of the total bone length residing along the control and SIT implant surfaces, respectively, at 3 weeks (Figure 7). After 12 weeks, the new bone accounted for 35% and 55% of the total bone length residing along the control and SIT implant surfaces, respectively (Figure 7). While the bone growth down the SIT implant surface was greater than that down the control implant surface at both time points, a difference in the extent of radial bone growth between the control and SIT implants was less evident. Qualitative results suggest that over the same time period, bone grows similarly around the SIT implant compared to controls.

### 3.3. Scanning Electron Microscopy

**Figure 8:**
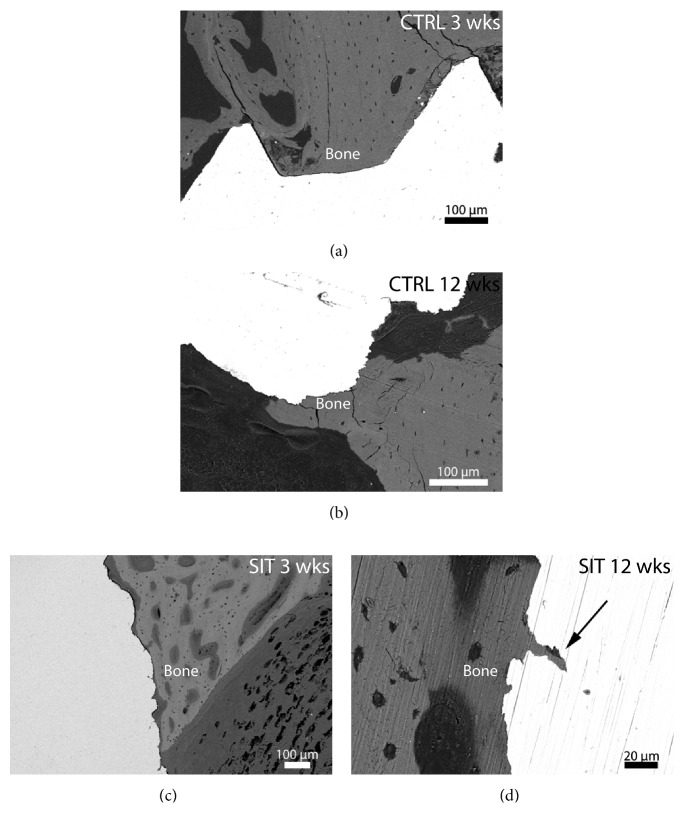
BSE-SEM images of the bone-implant interface after 3-week (a, c) and 12-week (b, d) implantation for (a, b) control and (c, d) SIT devices. Bone conduction along and into the microporosities of the SIT implant was observed after 12 weeks (arrowhead).

Similar to the micro-CT results, SEM images did not show a trabecular bone transition underlying the cortical bone, which indicated some misplacement of the implant off the target anatomical position. As such, bone contact was only possible originating from the cortical bone crest. Imaging of the embedded sections with SEM enabled qualitative assessment of the bone-implant contact in this cortical region. Three weeks after implantation, the cortical bone was present within the threads of the control implant (Figure 8). This mechanically interlocked bone was in contact with the control implant, while the new bone further down the length of the implant was primarily not in direct contact. However, stress cracks were observed at the mechanically interlocked thread tips. In contrast, little to no bone was in contact with the SIT implant after 3 weeks (Figure 8). The absence of threads also indicates a lack in mechanical interlocking. The bone structure around the SIT implant specimens appeared less developed with more porosity and randomly oriented osteocyte lacunae; however, in some cases, new bone formation was observed in between the stems of the SIT implant. Twelve weeks post implantation, the bone surrounding both implant types was more developed and in greater contact with the implant surfaces (Figures 8 and 8). The arrow in Figure 8 points to bone growth within the microporosities of the SIT implant suggesting improved osteoconduction. As with the 3-week samples, stress cracks were also present within the bone from the 12-week control implant.

### 3.4. Transmission Electron Microscopy

**Figure 9:**
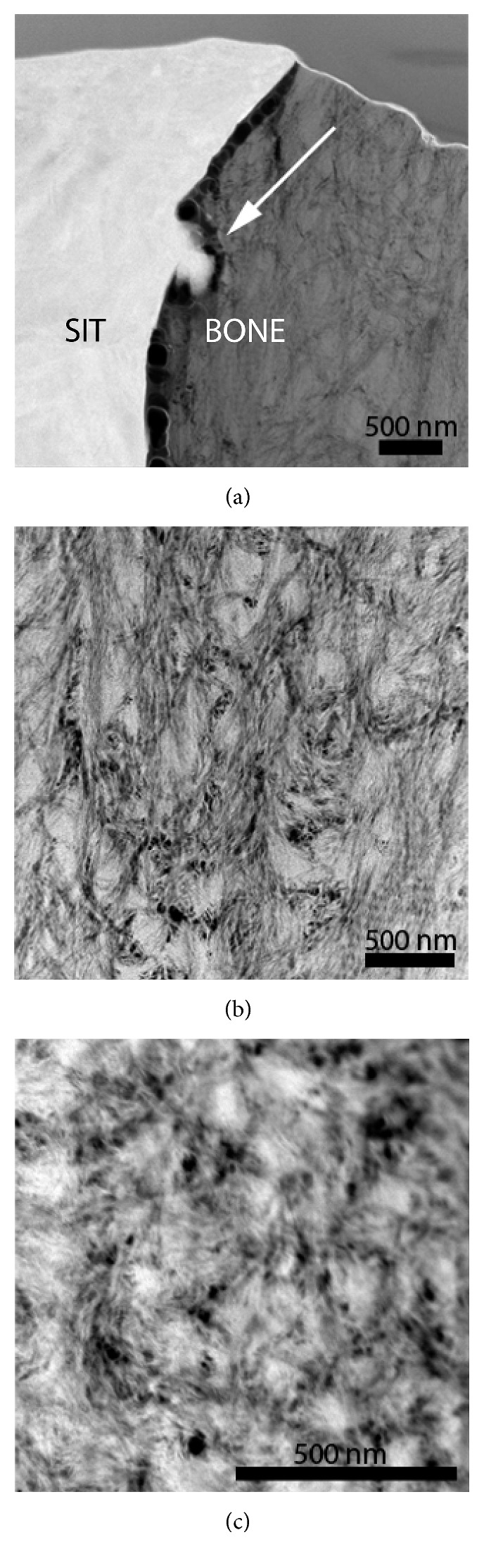
HAADF STEM images of (a) the SIT implant-bone interface after 12-week implantation. Bone growth around the nanoscale features was observed (arrow). (b) Ordered collagen fibrils, representative of mature bone, are noted adjacent to the implant at 12 weeks, while at 3-week implantation (c) partially disorganized collagen fibrils, representative of newly developing woven bone, are noted adjacent to the implant, consistent with the new bone structure.

To fully assess the osseointegration between the bone and implant and the quality of bone tissue at the interface, higher spatial resolution than that achieved by SEM is required.  Figure 9 shows a high-angle annular dark-field (HAADF) image of the SIT implant-bone interface after 12 weeks of healing. A separation at the bone-implant interface, likely due to mechanical stresses during removal and resin infiltration, was exaggerated by the FIB during TEM sample preparation. However, the matching contours of the bone and implant surface indicate that the implant and bone were likely in complete contact prior to retrieval. Preparation of a TEM specimen for the SIT implant at 3 weeks was not possible because of a lack of contact at the bone-implant interface. However, a specimen of the bone near the implant interface was removed for TEM. The difference in bone quality near the implant surface at 12 and 3 weeks is shown in Figures 9 and 9, respectively. The collagen fibers of the 12-week bone are more organized compared to the woven collagen fibers and visible mineral clusters of the 3-week bone, highlighting the differences in bone maturity.

## 4. Discussion

A novel implant design by Stemmed Implant Technologies Inc. is marked by a significant geometrical change that employs dual prong-like stems when compared to conventional implants that are generally conical threaded screws. The unique shape of this implant was achieved by the layer-by-layer, bottom-up approach of DMLS. It has been proposed that the new SIT implant will better resist rotational forces experienced during mastication and bruxism, as well as reducing the amount of bone removed and damaged during surgery and insertion. However, it is important to note that the authors did not evaluate any of these claims in this study. This pilot study aimed to understand the bone-implant interactions of the SIT implant and to predict its potential success in clinical implant scenarios. This was conducted with histology, X-ray, and electron microscopies. While the nature of a pilot study limits this work to a small sample size, the reported findings provide an initial assessment of the biocompatibility of the SIT implant and demonstrate its potential for further animal and clinical studies.

Histological analysis showed active bone growth and remodeling for both the SIT and control implants via osteoclast- and osteoblast-mediated bone matrix resorption and deposition. These observations are similar to previous histomorphometric evaluations of other DMLS implants placed in both sheep and humans [25, 26]. Early bone formation is evident by the presence of osteoblasts connected to the newly formed bone (Figure 4). The observation of bone debris for the control implant at 3 weeks and predominant osteoclast activity at 12 weeks compared to the SIT implant suggests a potential difference in the rate of bone formation and remodeling between the implants. Bone debris in the peri-implant space at early healing time points has been observed previously with threaded implants [26, 27] and may lead to delayed bone formation compared to implants devoid of threads [28]. Histological analysis indicates that the SIT implant shows comparable osseoconduction to conventional implants after 12 weeks, marked by complete bone encasement and active remodeling.

The nondestructive basis of micro-CT has been demonstrated as a useful tool for visualizing the entire implant and bone volume in two and three dimensions. Contrast gradients enable differentiation of new bone from old bone and identification of active remodeling sites, as well as sites lacking bone and osseointegration. These micro-CT results indicate that the implants were only anchored in the cortical bone, despite the usually large amount of the trabecular bone present in the metaphysis of rabbits where implants were placed, indicating a potential misplacement. Thus, this study is limited to the evaluation of cortical bone only. A fairly small amount of bone growth was conducted from the cortical bone crest down the implant length for the control implant compared to a slightly larger amount on the SIT implant that was even clearly visible between the implant stems at 3 weeks. Ideally, placement in the trabecular bone would maintain bone trabeculae between the stems for added stability. The reason for the observed difference in bone growth is somewhat unclear due to the potential interplay between differences in both the osteoconductivity of titanium and steel and the radial bone growth required for the control implants due to the threaded design. By three weeks, the majority of the bone volume, which was to encapsulate the implants, had been deposited and was remodeled into more mature dense bone, but not a greater quantity, by 12 weeks.

While micro-CT is ideal for a general overview of bone growth, it lacks the resolution necessary to visualize submicron features at the bone-implant contact. The greater extent of bone growth from the cortical region down the implant surfaces for the SIT implant was confirmed by SEM. The SIT implant conducted bone growth along its length and within the stems (Figure 8). The control implant initiated a limited amount of new bone formation, and large cracks were present within the cortical bone, perhaps caused by overtorquing during implant placement. Very tight integration, with no separation, was seen around the SIT implant, as the bone had grown into the micropores of the implant surface, which is an indicator of biocompatibility. Comparison of the bone-implant contact across studies remains challenging because of a lack of standardized methodologies employed to model bone growth and measure the bone-implant contact (BIC). Animal model selection, bone type, surgical procedure, heal time, sample preparation methodology, and selected implant length for BIC measurements varies across studies, all of which can influence the BIC [16, 26, 29, 30]. Nevertheless, previously reported BIC measurements of machined and analogous 3D printed implants after 2 weeks were 20% for both implant types [26]. In this study, we instead looked at the conduction of bone growth down the implant surface, since it was placed primarily in the cortical bone, and found as expected that the titanium SIT implant was a better conductor of bone growth at both early and late time points.

Due to bone-implant interface separation caused by FIB sample preparation, the exact integration between the bone and the SIT implant could not be analyzed; however, the maturity of the bone surrounding the implant could be evaluated to demonstrate the success of bone growth at the implantation site. TEM imaging of the lift-outs revealed differences in the orientation of the collagen fibrils and the presence of mineral clusters after 3 and 12 weeks. This suggests that the mechanism of distant osteogenesis is occurring during healing after the insertion of the implants. In distant osteogenesis, mature bone acts as a substrate for osteogenic cells to form a matrix that gradually encroaches upon the implant surface [31].

This pilot study was limited to an investigation of the structural and biochemical interaction of the implant device in vivo via advanced imaging modalities. To further validate these results, future work should focus on determining the mechanical integrity of the bone-implant interface. Mechanical testing of the implanted devices would also be beneficial to improving the understanding of the overall system and the potential advantages to using additive manufacturing as a production method for dental implants. This could potentially be completed in vivo through methods such as resonance frequency analysis to determine implant stability [32] and via pullout tests to confirm adequate mechanical strength [33]. Complementary information from in vitro testing, such as investigating cell viability [34], may provide additional insight into the biocompatibility of the device. Other works reporting 3D printed implant devices have shown promising cell viability and biocompatibility [35–37].

## 5. Conclusions

Additive manufacturing provides a means for innovative dental implant designs with inherent surface features which facilitate bone integration. Initial observation of a dual-stemmed 3D printed dental implant has shown successful bone growth and bone-implant contact similar to conventional and other 3D printed implants up to 12 weeks of healing in rabbits. In some cases, new bone formation was noted in between the stems of the device, although the stems were not within a trabecular bone region. Conventional implants showed mechanical interlocking but did have indications of stress cracking and bone debris. This pilot study demonstrates that this 3D printed implant design is biocompatible, as it allows for successful osseointegration in rabbits up to 12 weeks, and supports additional studies to obtain more statistical validation, including mechanical testing.
